# Emergent Behaviors in a Bio-Inspired Platform Controlled by a Physical Cellular Automata Cluster

**DOI:** 10.3390/biomimetics1010005

**Published:** 2016-08-31

**Authors:** Tareq Assaf, Richard Mayne, Andrew Adamatzky, Chris Melhuish

**Affiliations:** 1 Bristol Robotics Laboratory, Bristol BS16 1QY, UK; Chris.Melhuish@brl.ac.uk; 2 Unconventional Computing Centre, University of the West of England, Bristol BS16 1QY, UK; Richard.mayne@uwe.ac.uk

**Keywords:** cellular automata, emergent behavior, bio-inspired robotics, morphological computation

## Abstract

This work illustrates behavior patterns and trajectories of a bio-inspired artificial platform induced by a cellular automata (CA)-based control strategy. The platform embeds both CA control as physical electronic architecture and a distributed hardware layer as effectors. In this work, we test both the functionality of the novel hardware’s components as well as the device’s capabilities in locomotion tasks. We also observe the trajectories and patterns emerging from different initial states of the CA excitation and hardware configurations. Two main result sets emerge from this study: the first set illustrates different trajectories according to different initial excitation of the physical CA controller layer. The second set suggests the potential of the developed platform for generating complex patterns of control, as well as indicating emergent characteristics similar to those common to morphological computation approaches in generating localized perturbations without affecting or notifying the central controller.

## 1. Introduction

This work exploits a newly developed bio-inspired artificial platform prototype that implements a physical cellular automata (CA) [1] cluster as control layer. A CA is a simple mathematical representation of a complex system consisting of a homogeneous grid of cells which may assume a finite number of “states” (corresponding to excitation of a node) which evolve in discrete time based on the states of its neighboring cells according to a predefined rule. CA have been demonstrated to exhibit exorbitantly rich dynamics in their modeling of a wide range of biological, chemical and physical phenomena; we refer the reader to [2] for an overview of the topic.

Emergent locomotion behaviors generated by different initial excitation conditions of the CA cluster and hardware configurations in our platform are observed and discussed here.

This work is part of the Leverhulme Trust funded project entitled “Towards Artificial Paramecium”: the project aims to design, develop and investigate a physical architecture of CA capable of controlling a distributed multi-actuator-sensory array by merging CA control strategy and hardware effectors, inspired by the cilia of the unicellular protist *Paramecium caudatum*.

This micro-sized protist is covered with thousands of cilia, which are finger-like membranous organelles that beat rhythmically, affording the organism a means for locomotion, obstacle avoidance and food gathering. Cilia are also found within other microscopic [3] and complex organisms, including the bronchial epithelia of mammals, where they drive the mucociliary escalator [4]. Their collective movement can generate several patterns in order to achieve tasks additional to locomotion, such as parallel manipulation; metachronal (sequential) waves are an emergent phenomenon instantiated in cilia arrays towards achieving these tasks. The extreme decentralization and control robustness shown by the microorganism has been taken as a source of inspiration. An artificial counterpart with similar characteristics and capable of emulate CA control strategies has been designed and implemented in a small scale.

**Figure 1:**
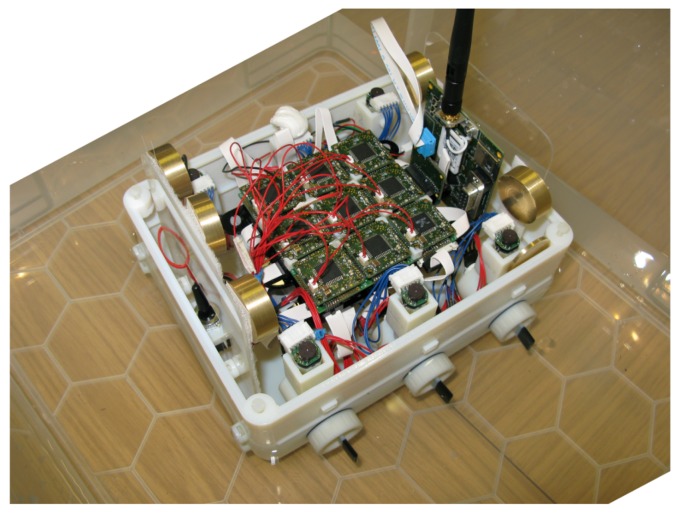
Physical platform. The physical cellular automata (CA) architecture is connected to six independent paddlers provided with Encoder and index hall effect sensor. It is powered by a three-cell lithium battery.

Figure 1 shows the prototype platform used in this work, which constitutes two main components: the CA physical modular cluster layer and the hardware layer (effectors and sensors). The hardware layer can be tailored and designed to fit different purposes according to the application. We will refer to the controller layer, although constituted of a modular electronic hardware, as CA physical layer or cluster in order to avoid confusion between the two components.

The objectives of the work presented are: (i) to test the novel bio-inspired platform in locomotion tasks; (ii) test the CA physical architecture control combined with physical system; and (iii) observe emerging behaviors from different CA initial configurations and hardware conditions.

To these ends, the work describes the overall platform, the experimental settings and the initial excitation of the CA layer used.

The paper is structured as follows: Materials and Methods (Section 2) reports the background, review of previous works and presents the different platform components. The experimental environment, and an overview on the data processing used to obtain the trajectory and the experiments are also described. Section 3 and Section 4 are illustrates and discuss the results, respectively. Conclusions, and comments to the work and results are presented in Section 5.

## 2. Materials and Methods

The CA control technique is a powerful control tool used in many fields including: swarm robotics [5,6,7,8,9], data processing [10,11,12] and behavior control [13,14,15,16] to name some examples.

Those examples illustrate the flexibility and adaptability of the CA to solve complex problems. The CA is, however, normally run within simulation environments that can simulate a large number of cells, rules and interactions quickly and efficiently by using standard personal computers (PCs). Such approaches, although highly effective, might present technical difficulties if used to control a large number of actuators and sensors. The centralization can only emulate parallelism and often needs to rely on subunits to control the individual effectors/sensors.

In order to find alternative solutions to this, works have been focused on the development of physical CA solutions both based on single chip solutions [17,18,19] and physical lattices in order to move and sort objects or generate haptic feedback [20,21,22]. These approaches are both centralized and semi-decentralized.

Previous works have also been focused on similar topics and strategies for generating a distributed lattice to manipulate objects [23,24,25]. The focus of those works was the propulsion of objects placed on the lattice and the manipulation of them by means of traveling waves generated by vibrating motors controlled by CA excitation.

The platform developed and exploited in this work is based on complete decentralization of both CA and low level control. Each module is a single cell of the CA and it is physically interconnected with its neighbors. Although this single, fully decentralized cell approach is comprehensive of all disadvantages affecting modular designs, it allows for scalability and flexibility. Parallelism, fine low level control, physical interconnection, scalability and flexibility features are different from the semi-decentralized approach (e.g., single module controlling single or multiple actuators and simulating larger number of CA cells). Each module in this architecture retains the capability to simulate a larger number of cells if needed.

### 2.1. Control

**Figure 2:**
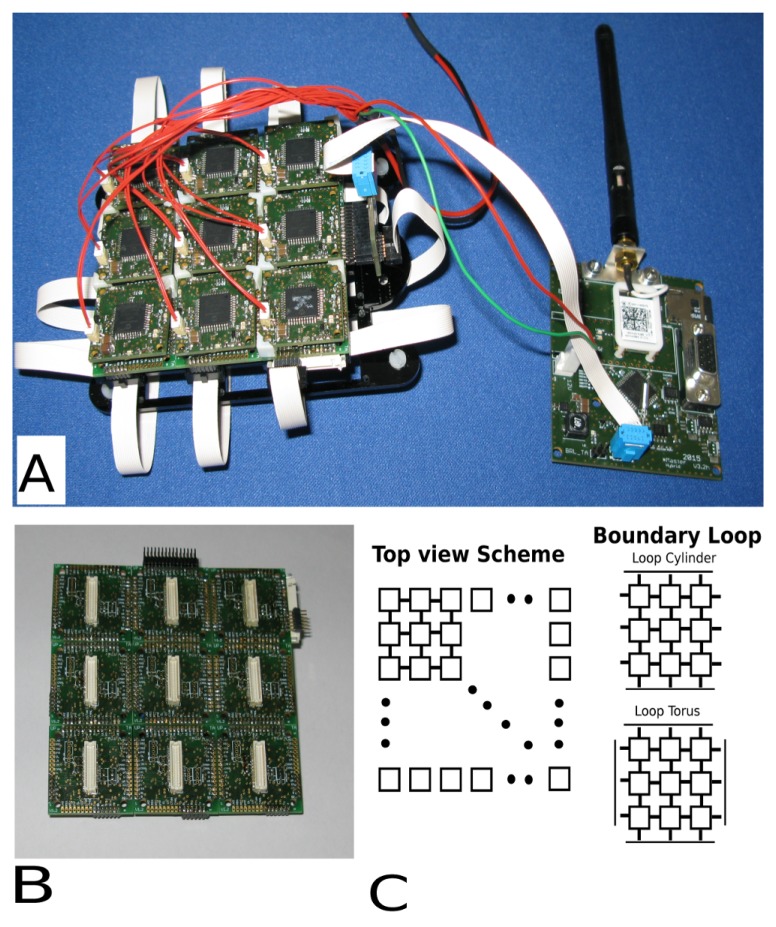
Image illustrating the physical 3×3 CA network and the coordinator wireless board. Also visible are the ribbon cables used as boundary loops (**A**). The connection boards (**B**) and the connection schemes (**C**) are also presented.

As mentioned previously, the artificial platform is constituted by both the CA control layer and the hardware effectors. In this early stage prototype, the CA control cluster has nine cells. The state of each cell is determined by the state of each of its eight neighbors (although the CA may be run with either Moore or von Neumann neighborhood). The CA layer is illustrated in Figure 2A, with connection boards (Figure 2B) laying underneath the CA modules.

Figure 2C illustrates the array concept, interconnections and boundary loops. Each cell-to-neighbor connection is physical. The edges of the cell array can be left disconnected or connected to the other cells (normally to the opposite edge) obtaining, in this way, two distinct behaviors. In the first case, the CA rules will dissipate on the edges of the array; in the second case, the CA rules will continue as in a media without borders. In the example reported, the media can be both a cylinder or a torus, geometrically speaking, virtually wrapping the planar structure on its own.

The boundary condition set for this study is the torus in which the edges are connected via a ribbon cable, as illustrated in Figure 2A.

In this study, we used a three state rule as illustrated in Equation (1), where *k* is equal to the number of neighbors (e.g., k=8 or k=4). $x^{t}$ is the current state of the cell and $x^{t+1}$ is the next state. $\sum \eta_{i}^{t}+$ counts up if the state at time *t* of the *i*th neighbour (*η*) cell is excited (+).

(1) $$\begin{array}{c} x^{t+1}=\left\{\begin{array}{cc} \mathrm{Excited}\;(+) & \mathrm{if}\;x^{t}=•\mathrm{and}\sum_{i=1}^{k}\eta_{i}^{t}+\geq 2\\ \mathrm{Refractory}\;(-) & \mathrm{if}\;x^{t}=+\\ \mathrm{Resting}\;(•) & \mathrm{otherwise} \end{array}\right. \end{array}$$

A resting (•) cell becomes excited if at least two of its neighbors are excited. An excited cell becomes refractory (−), and a refractory cell becomes resting regardless of the states of its neighbors; these transitions are unconditional.

This rule can generate traveling waves and other patterns within the boundary condition we chose.

The three states of the CA influence a specific behavior of the end effector as follows:

- Excited : Motor ON, positive direction, fixed motor speed proportional-derivative (PD) control
- Refractory : Stop motor motion, motor OFF
- Resting : No Actions

The positive motion direction of the motor and speed are two variables that can be configured at start up or modified during run time for each individual cell. Such change can be performed locally or from the central node, which is the network coordinator.

The frequency of data polling for the platform for this specific experiments has been 50 Hz with a variable CA update frequency ranging from 1 to 5 Hz depending on the experimental conditions.

### 2.2. Platform Actuation and Characteristics

**Figure 3:**
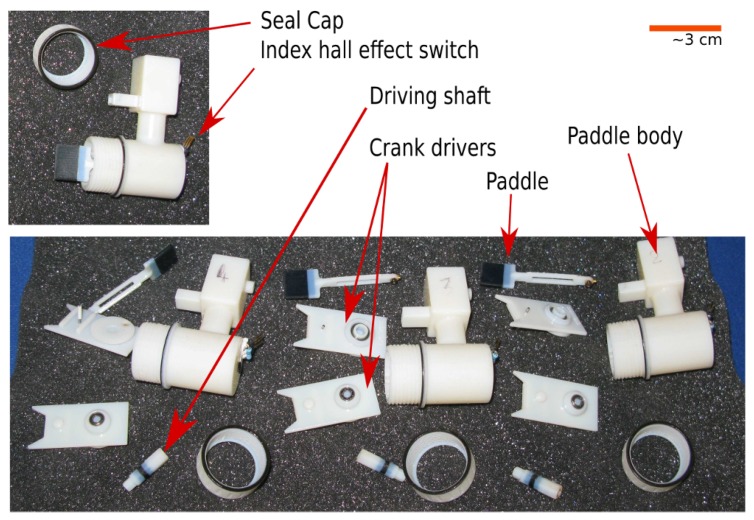
Photograph illustrating the six paddle components and their assembly. Each paddle is a simple mechanical crank powered by a micro-motor (90 mW).

The effectors chosen to address the locomotion task, and mimic in a different scale domain the cilia, are paddles. Although parallel manipulation is also one of the main goals of the project, locomotion has been chosen as case of study for this test-run because: (i) it requires a smaller number of CA cells to be performed and; (ii) locomotion in this study can be considered as a parallel manipulation in which each individual paddle influences the environment by displacing a small amount of water and hence contributes towards the overall momentum of the entire body, such that traveling waves can be collectively generated. At this stage no external sensors are used or implemented. The paddles are illustrated in Figure 3. The paddle is a 3D printed structure which is comprised of five elements including the rotating crank to turn the motor torque output into the paddle stroke. Each paddle is powered by a micro-motor and has intrinsic differences compared with the others including top speed, friction and starting torque/current.

The design is able to be mounted on the frame and ensures water resistance. The water will fill the crank chamber reaching up to the motor shaft above the buoyancy line. This design also allows us to reduce the complexity of the system as paddle/motor junctions do not need to be fully waterproof.

The overall weight of the platform is approximately 1.1 kg. Its shape (see Figure 1) is a rectangular box. While this is not an ideal boat profile, it was chosen for its simplicity, stability and the ease of accommodating the hardware within. The overall weight and hull shape compared with the paddle number, size and motor power reduce the reaction time of the platform to individual paddle action. This is both an advantage to overcome some inconsistency in the paddle trust but it makes it difficult to change direction of the platform once a certain amount of momentum has been built up. The platform, however, has been conceived for low speed and low energy actuators, and such a problem was foreseen and accepted.

In order to solve the problems arising from the weight to propulsive power ratio, a water environment was selected (see following section). However, the platform is subjected to perturbations due to water waves and ripples.

### 2.3. Environment

**Figure 4:**
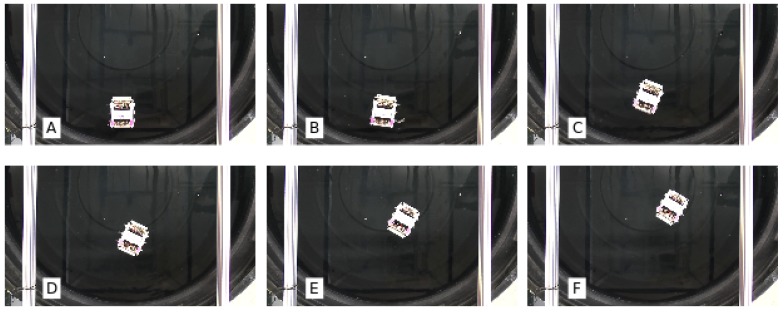
Six snapshots captured from a video recorded during an experiment show the trajectory of the robot within the swimming pool environment. (**A**) Initial position; (**F**) Final position.

All experiments were carried out in a small swimming pool of 2 m diameter. A camera placed above the working area was used to record the robot in action; Figure 4 reports an experiment in situ.

During each experiment two sets of data were collected: a video was recorded from above in order to be post-processed and obtain information on the robot behavior; the robot stored a set of information about the run locally on a Secure Digital (miniSD) card. This information included the CA state for each cell, motor current position and velocity.

### 2.4. Data Processing

The video recorded during these experiments was post-processed using OpenCV library in order to generate a trajectory path. Each frame from the videorecording was analyzed, and the trajectory and orientation of the robot were calculated as follows:

- Trajectory and direction:
  1. Frame to grey scale
  2. Grey scale erosion and dilation to remove noise
  3. Edge detection on clean image
  4. Find bigger blob, and calculate center of mass and direction 0–180 degrees
- Orientation:
  1. Frame to color filter
  2. Color filtered center of mass used to discriminate the orientation 0–360 degrees

The data stored locally contains information that can be used to reconstruct the CA evolution and check the platform parameters after each run if needed. In this work those data are not relevant and some of them were only used to check if the CA was executed correctly.

### 2.5. Experiments

This subsection details and explains the experiments performed, the settings used and expected outcomes. We focused on four topics, which are listed below:

- Assessment parameters: Platform repeatability, robustness and reliability in the following three experimental setups
- Experiment 1: After choosing a set of 11 initial CA conditions, the resulting platform trajectories and behavior were observed; this was performed at a fixed update frequency of 1 Hz
- Experiment 2: Three initial conditions with the most distinguishable trajectories have been used. In this experiment the update frequency of the selected CA initial conditions was tuned between 1 and 5 Hz in order to observe for any effects on platform behavior
- Experiment 3: A single initial CA condition was selected and one parameter of the platform effectors was tuned in order to observe what changes this might have caused

**Figure 5:**
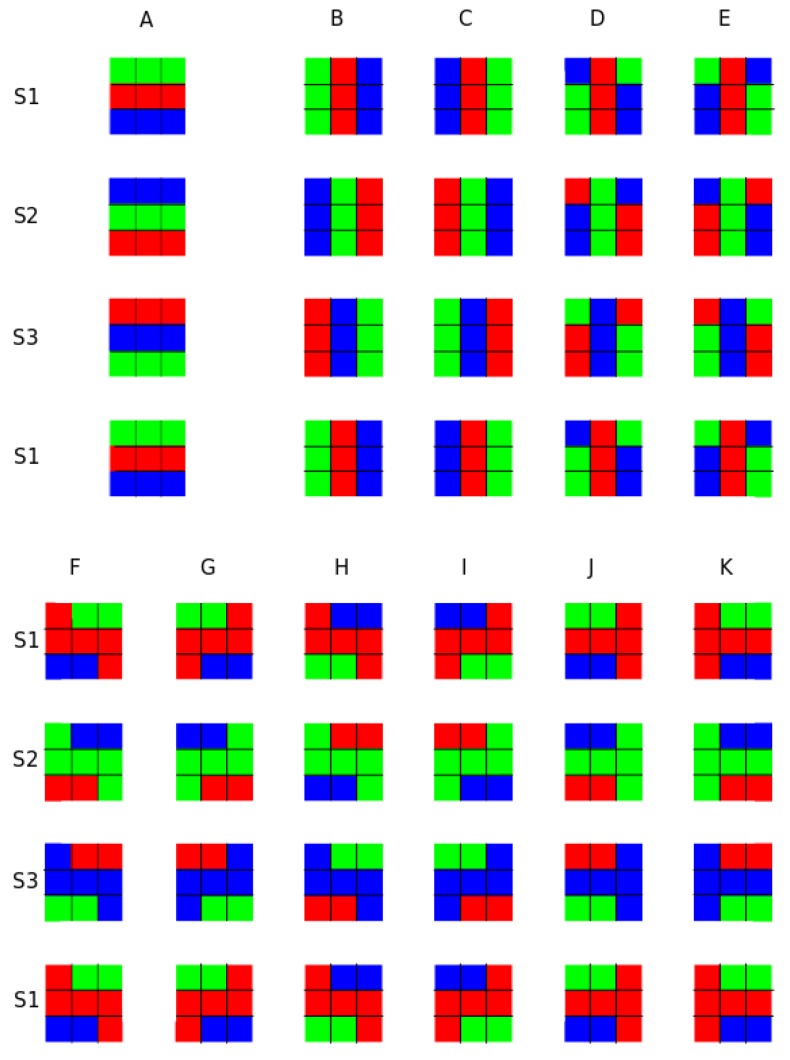
Configurations of the 11 initial CA conditions (A–K) and their evolutions (S1–S3) used in this work. Green, blue and red correspond to the CA states: excited, refractory and resting states, respectively.

The initial conditions used in this work are summarized and illustrated in Figure 5.

Figure 5A–K show the 11 initial CA conditions chosen, and illustrate the excitation dynamics and evolution cycles (S1–S3) of all initial conditions. Each configuration has a cycle period of three steps. Green, blue and red correspond to the CA states (i.e., excited, refractory and resting states, respectively). The excitations depicted in Figure 5 A–K are used in Experiment 1. Excitations A–C are used in Experiment 2. Excitation A is used for the last experiment.

In order to evaluate the trajectory and locomotion behavior of the platform, the center of mass of the robot was calculated and used to determine its position in the camera field of view. Markers on the platform body (aft position) were used to calculate the orientation of the platform. These two pieces of information were directly extracted from the video captured during the experiments in post-processing. Every experiment lasted for roughly 20 s—time required to move from the starting position to the edge of the camera field of view during a linear trajectory. The initial configuration, as shown in Figure 5A, also produced a symmetric metachronal wave traveling along the robot.

No external sensors were used in order to both reduce the complexity of the evaluation tests and observe the platform in open-loop behavior.

The platform in its current iteration uses internal sensors to control the actuators. This implies that the trajectories are subjected to drifts caused by water ripples due to movement and non-stationary conditions of the water surface. Another source of noise on the trajectory is due to the differences between actuators’ manufacture and characteristics. In the Results and Discussion (Section 3 and Section 4, respectively), the implications of these sources of noise are further detailed; however, in this study such disturbances do not seem to have a major impact upon the observations and the results presented here.

## 3. Results

This section reports the observations and data collected during Experiments 1–3.

### 3.1. Experiment 1 — CA Initial Condition Excitation

**Figure 6:**
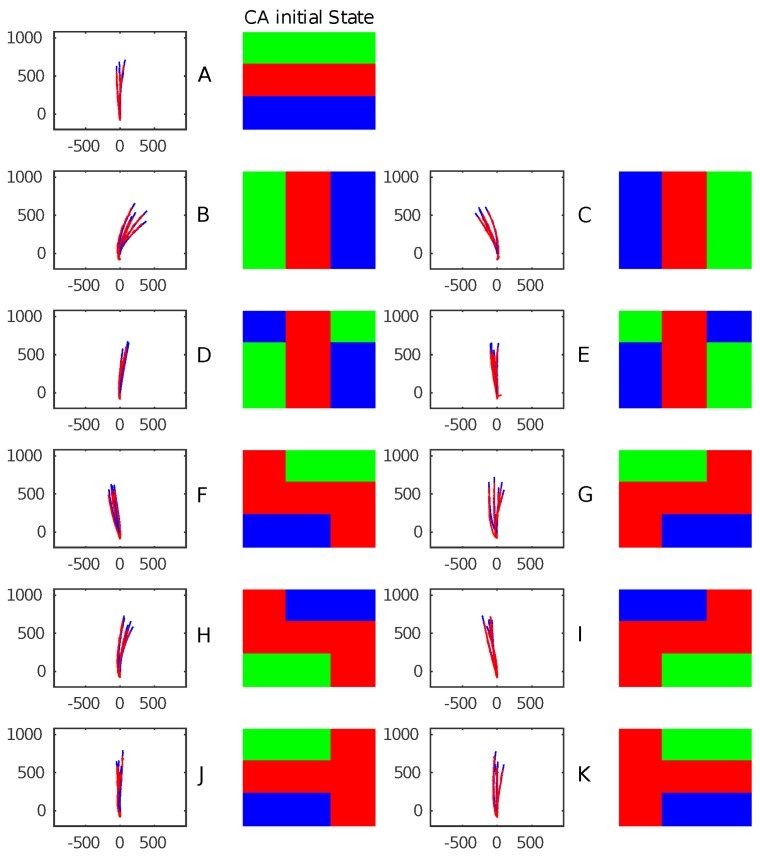
Plots showing five trajectories of the platform for each initial CA condition tested (A–K). The trajectories in the camera’s field of view are shown in pixels.

The first experiment focuses on passively observing the trajectory and behavior resulting from generating predefined initial excitations of the CA layer. Figure 6 illustrates the different 11 initial CA conditions (A–K) and their resulting trajectories obtained in two experiment sessions. A total of five trajectories are overlapped. The CA update rate was 1 Hz and the paddle target speed for the PD controller embedded within the modules was three turns per second.

Each initial condition generates a specific pattern of trajectories. This is particularly visible in the first three conditions. Each excitation results in the activation of all paddles with different timing and sequences. This mostly affects the initial orientation and stabilizes towards a linear trajectory when momentum is built. The other causes of trajectory and pattern alterations are due to external forces (e.g., water ripples).

Due to choosing a random starting point and angular orientation of the robot, trajectories are represented as the origin and rotated according to the initial orientation calculated as the average of a few initial frames.

This method does not remove or counteract most of the noise induced by the external environment, but renders the trajectory simply to display and analyze.

### 3.2. Experiment 2 — CA Frequency Tuning

**Figure 7:**
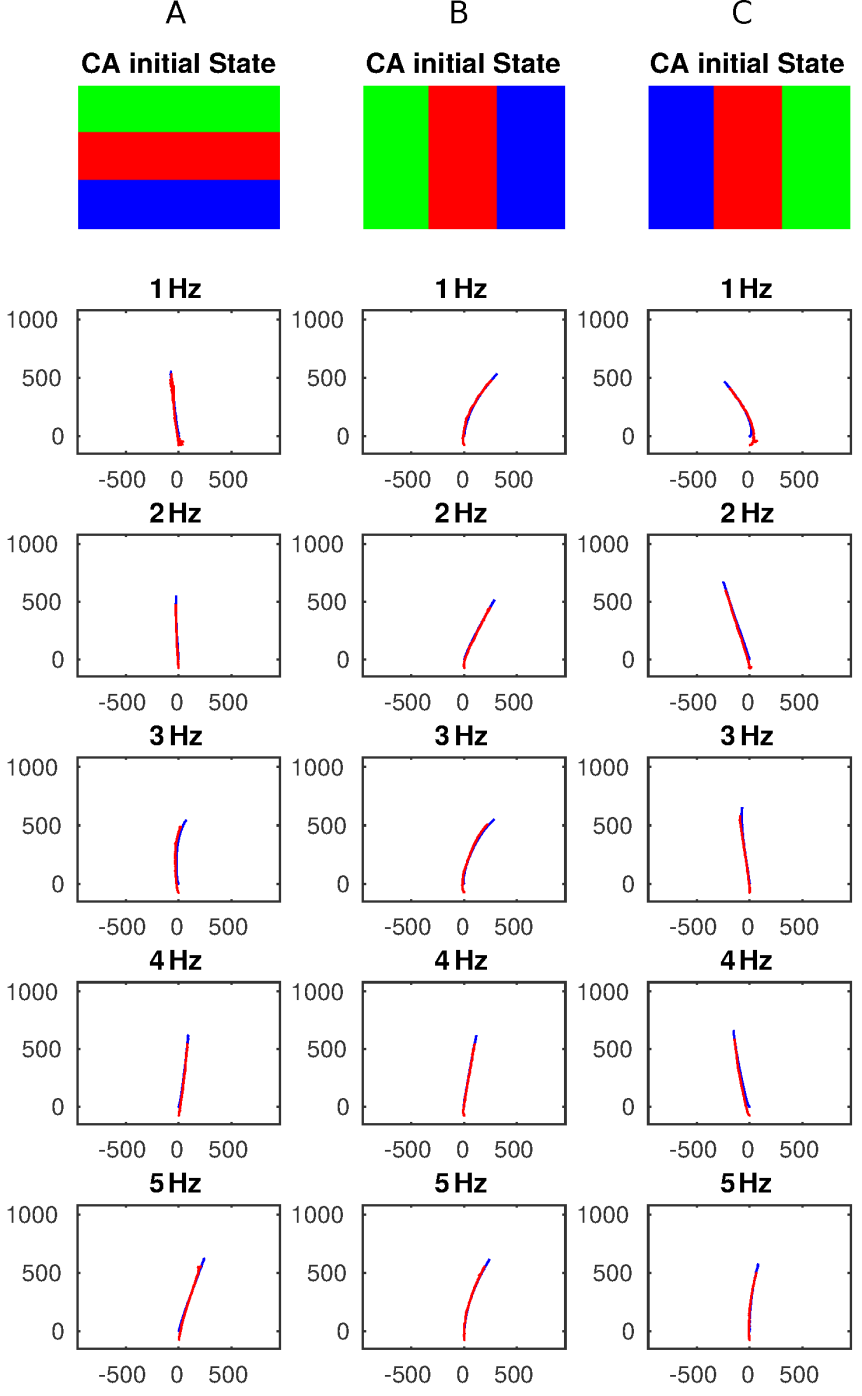
Plots illustrating the three initial CA conditions (A–C) used and the resulting trajectories obtained by changing the CA update frequency from 1 to 5 Hz. All the trajectories are shown in pixels.

In the second experiment, the CA update frequency was changed from 1 to 5 Hz in graduations of 1 Hz. The initial CA conditions that generated well-defined and distinct trajectories have been selected and used (conditions A,B,C). The results generated are shown in Figure 7. For each of those three conditions the CA update frequencies were tested. No significant changes in trajectory were expected; however, the results show that at frequencies of 1–2 Hz, the PD control can efficiently control the overall speed of the paddles but at higher frequencies (3–5 Hz), this cannot counteract some of the intrinsic differences of the actuators including friction, inertia, and start up current/duty cycle that might affect the final trajectory. Therefore, at higher update frequencies the platform tends to generate trajectories sensitively reflecting mechanical imbalances in the system. In this output example, it turns slightly to the right and all three initial conditions generate a similar behavior. This result is subjected to changes in actuation performances, as the set of results presented here are taken all in one session, and we assumed that the mechanical characteristics remain constant. Changes in those characteristics (e.g., internal friction) of the paddles due to friction or other mechanical issues would result in a different trajectory, in particular at higher frequencies. The simple robot shape and imbalances of the flow create a chance of deviation to one or other side.

This might be remedied, in our opinion, if external sensors, improved mechanics and/or close loop control could be exploited. At the same time, it also represents a feature showing that the platform behavior can change by chaining the CA update frequency. For example, the third configuration generates a left, straight and right trajectory at 1 Hz, 3 Hz, and 5 Hz, respectively. Although this result depends on the intrinsic manufacturing and characteristics of both paddles and motors and, therefore repeatability is not guaranteed and further investigation is required, it underlines that a distributed physical CA platform combined with a physical actuator layer can exploit local differences to generate a global behavior just by tuning a single parameter. At lower frequency, as depicted in Experiment 1, the velocity control contributes to compensate the mechanical differences (if not too great). Therefore, this is also proof of some robustness in dealing with differences in mechanical components.

### 3.3. Experiment 3 — Modulating Hardware Parameter

**Figure 8:**
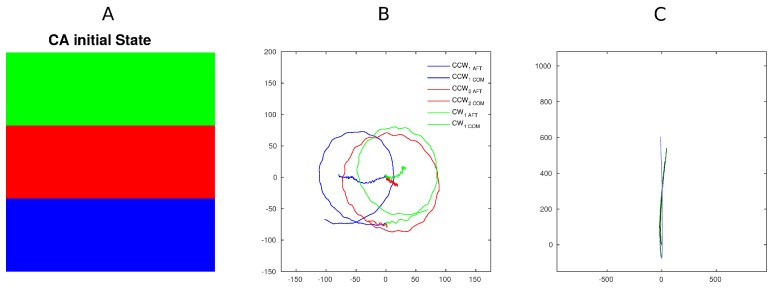
Image illustrating the two emergent behaviors generated by tuning how the motor would execute the same CA command (initial condition, A). In the first case (B), the platform rotates on its own (positive direction for all motors was counterclockwise (CCW 1,2) or clockwise (CW 1)). In the image, the trajectory followed by the center of mass (COM) and the aft of the platform are visible. In the second case (C), the positive direction was properly set to obtain forward motion (CCW left paddles or CW right paddles). All the trajectories are shown in pixels.

The last experimental setup illustrated focuses on changing a platform parameter, in particular, the positive motor spinning orientation. A single excitation was used. Figure 8 compares the two behaviors. In the first excitation pattern, all motors’ positive rotation was counterclockwise (CCW—left side) or clockwise (CW—right side), therefore generating a forward traveling wave (as in the previous experiments). In the second excitation pattern, all motors were set to turn as positive to CCW or CW. This modification generated revolutions around the centre of mass of the platform by using identical excitation patterns. Although this behavior was expected due to the simplicity of the experiment, the result implies that radically different behavior can be triggered by local sensory information without effecting central control. In other terms, if other variables were tuned locally (e.g., speed) by local sensors on the single unit/CA cell, this would, in our opinion, generate a different behavior. Such changes can be dealt with both, at control level by tuning and altering the CA rule locally or locally by the low level control.

## 4. Discussion

The idea and approach outlined in this study demonstrate good results in terms of overall platform capabilities. The performances and outcomes of this CA platform are a promising basis towards enlarging the number of CA modules towards a manipulation lattice such that large-scale simulated ciliary arrays may be fabricated. Although the experiments detailed here only cover a small-scale study of open loop trajectory behavior evaluation, the electronic modular structure is capable of sustaining different CA rules and expected degree of control. The computational power on board of each cell allows for even more complex and multi-layered CA, or other computing strategies running simultaneously. This provides enormous freedom and flexibility to the end-user to plan, develop and analyze a broad range of controls and applications scenarios.

The modular nature of the entire platform allows the user to change single components such as effectors, sensors or CA module topology in order to obtain new or extend existing capabilities.

The results presented show how this physical CA controls multiple actuators and modulates the output behavior. In particular, the examples presented by using this small section of CA array can generate a consistent number of different scenarios. Other rules can be implemented and connections between neighbors can be both removed or extended (virtually forwarding distant module states). The initial excitations presented here represent a first set of elementary building blocks toward more complex control strategies.

In this work we also tuned a few of the possible variables involved, more specifically the update frequency of the CA network and the motor behavior (motor positive rotation convention) to the same control command. These two variables and the initial conditions represent three characteristics that can be tuned both at start up (as in this work) or during run-time. A few examples of other possible tuning parameters and their combinations are listed below:

- Different CA rules
- Paddle velocity profile
- PD or proportional–integral–derivative (PID) parameters
- Control strategies of the effectors
- External sensors to tune internal responses (e.g., motor rotation, speed or changes in CA module state)

The last result presented (observation of Experiment 3), in our opinion, is the most promising and significant towards interesting behavioral complexity and dynamic control that could be generated by using the platform presented (or larger array versions). The fully decentralized approach allows us to control singularly each component of the physical layer with both global control (CA) and local settings. The local setting can be driven by external inputs of specific CA states but most importantly altered by local sensor inputs. Although this envisioned scenario would significantly increase the complexity of the platform, it generates the opportunity to facilitate emerging behavior driven by both CA evolution and/or physical body interaction with the environment. The result presented shows the feasibility of such potentiality in one of the simplest cases.

## 5. Conclusions

The results presented illustrate how the platform developed might be used even in its early stage. Firstly, the custom developed CA physical electronics proved its endurance, thereby highlighting points of strength and weakness. Secondly, the CA control combined with a physical, simple, robot generated significant changes in behaviors, simply by tuning the initial conditions. Thirdly, modulating the initial conditions would generate, once triggered by internal and external inputs, more complex behaviors. Fourthly, the robot exhibited changes in behavior as another consequence of simple tuning of end effector response parameters.

This final result in particular opens interesting questions such as: how can identical command transmission (swap in frequency) or effectors response pattern generate/alter complex emerging behaviors? These are linked, in particular, with the results of Experiment 3, and belay what are probably the most promising characteristics of this platform. On one hand, the CA controller initial condition or update frequency can influence and tune the behaviors of the actuation layer and platform, and therefore generate a distributed control level strategy. On the other hand, the controlled (second) physical layer can be affected in a similar way, changing at run-time how a command is carried out or executed.

The CA control in this work is simple to both evaluate the platform performances and observe if and how simple modifications in conditions would have an impact, if any, on the output trajectories. In particular, Experiments 2 and 3 show how CA control is normally executed. It is not affected by the changes but the output trajectories and behaviors are. In the first case, the mechanical properties of the actuation layer take over the control and in the second case the behavior is induced by the interpretation of the control signal.

Altering the effectors response (in Experiment 3) to the same command intrinsically creates a middle “virtual” layer similar to local response that is capable of triggering behavior without notifying or involving the main control system. The control system might not even be aware that a perturbation occurred. This middle layer, although not intentionally designed, is directly linked with the distributed nature of the system presented.
